# Modification of the coronal plane alignment of the knee classification considering the joint line convergence angle in high tibial osteotomy: a geometric approach

**DOI:** 10.1186/s13018-026-06922-0

**Published:** 2026-05-08

**Authors:** Ju-Ho Song, Bum-Sik Lee, Jong-Min Kim, Seong-Il Bin, Jaejung Ryu

**Affiliations:** Department of Orthopaedic Surgery, Asan Medical Center, College of Medicine, University of Ulsan, Olympic-ro 43-gil, Songpa-gu, Seoul, 05505 Republic of Korea

**Keywords:** High tibial osteotomy, CPAK classification, Joint line obliquity, Joint line convergence angle

## Abstract

**Background:**

Excessive joint line obliquity (JLO) is associated with poor outcomes in open wedge high tibial osteotomy (OWHTO). The Coronal Plane Alignment of the Knee (CPAK) classification categorizes knee phenotypes by alignment and JLO but overlooks the joint line convergence angle (JLCA), which impacts JLO pre- and postoperatively in OWHTO. This study aims to modify JLO calculation by incorporating JLCA through a geometric approach, with the goal of better reflecting the knee joint orientation under weight-bearing conditions.

**Methods:**

323 knees that received OWHTO were retrospectively reviewed. The study included OWHTO patients who had pre- and 1-year postoperative long-standing hip-to-ankle radiographs for CPAK classification and JLCA measurement. JLOs were calculated using two methods based on a geometric approach: conventional JLO (cJLO), defined as (lateral distal femoral angle [LDFA] + medial proximal tibial angle [MPTA])/2–90°, and modified JLO (mJLO), which incorporates the JLCA, defined as (LDFA + MPTA)/2 + JLCA/2° − 90°. These were compared to the knee joint orientation using intraclass correlation coefficients (ICCs) and Bland-Altman plots. Clinical outcomes were evaluated using the Hospital for Special Surgery (HSS) score.

**Results:**

Mechanical hip-knee-ankle angle (mHKA) was corrected from − 7.1 ± 2.7° preoperatively to 2.1 ± 2.3° at 1 year postoperatively, and JLCA decreased from 3.1 ± 1.9° to 2.4 ± 1.7°. The HSS scores showed significant improvement, rising from 68.6 ± 11.7 preoperatively to 91.3 ± 7.4 postoperatively (*p* < 0.001). The knee joint orientation was measured at − 1.0 ± 1.9° preoperatively and 1.9 ± 2.3° postoperatively at 1 year. Comparatively, cJLO values were − 3.3 ± 1.5° and 0.9 ± 2.0°, while mJLO values were − 1.8 ± 1.8° and 2.1 ± 2.3°. ICCs showed preoperative and 1-year postoperative cJLO at 0.842 (95% CI, 0.804–0.873) and 0.927 (95% CI, 0.909–0.941), respectively, while mJLO demonstrated 0.881 (95% CI, 0.851–0.904) and 0.949 (95% CI, 0.936–0.959). Bland-Altman plots showed that both preoperative and 1-year postoperative mJLO had smaller differences from the knee joint orientation than cJLO, indicating that mJLO more closely reflects functional joint orientation.

**Conclusion:**

The mJLO, derived through a geometric approach that incorporates JLCA, was found to more closely reflect the knee joint orientation compared to the cJLO described in the CPAK classification. To effectively apply the CPAK classification in OWHTO, it is essential to account for JLCAs before and after surgery. Accordingly, mJLO can be represented as (LDFA + MPTA)/2 + JLCA/2° − 90°.

## Introduction

Open wedge high tibial osteotomy (OWHTO) redistributes the load on the medial compartment by changing the proximal tibia geometry [1–3]. The postoperative anatomic change of the proximal tibia can be represented by joint line obliquity (JLO), and an excessive increase in JLO has been reported to indicate poor outcomes in OWHTO [4–6]. When the orientation of the joint line deviates from its physiological state, shear stress on the knee joint may increase, potentially leading to poor clinical outcomes as well as chondrocyte apoptosis [7–9]. 

The Coronal Plane Alignment of the Knee (CPAK) classification is a simple and intuitive method for categorizing knee phenotypes based on overall alignment and JLO. In the CPAK classification, JLO is determined by the lateral distal femoral angle (LDFA) and the medial proximal tibial angle (MPTA) using an arithmetic method [10]. The CPAK classification can be effectively applied in OWHTO, which corrects coronal plane alignment. However, it does not take into account the joint line convergence angle (JLCA), which indicates chondral wear progression and changes in soft tissue tension around the knee joint [11–13]. Varus alignment arises not only from bony components such as the LDFA and MPTA but also from an increased JLCA [14–16]. 

Hsu et al. recently modified the original CPAK classification, noting that the arithmetic JLO is defined as half the sum of the LDFA and MPTA minus 90° [17]. However, this method also has limitations, as it relies solely on calculating the bony origin of varus in the femur and tibia, making it less applicable for patients with increased JLCA values in varus osteoarthritis. Therefore, this study aims to modify the JLO calculation by incorporating JLCA through a geometric approach, with the goal of better reflecting the knee joint orientation under weight-bearing conditions. The null hypothesis was that there would be no difference between the conventional JLO (cJLO) and the modified JLO (mJLO) in their agreement with the measured knee joint orientation, both before and after OWHTO.

## Methods

Patients who received OWHTO from January 2010 and to July 2021 were retrospectively reviewed following approval from our institutional review board. OWHTO was recommended for patients with persistent medial compartment walking pain that did not improve after three months of conservative treatment, particularly in cases of varus malalignment exceeding 3°–5° and predominantly originating from the proximal tibia. Contraindications included medial compartment arthritis of Ahlbäck grade ≥ 3, flexion contracture greater than 10° or advanced motion limitation, lateral compartment arthritis, and active inflammatory arthritis.

The inclusion criteria of this study were (1) primary medial OWHTO with locking plate fixation and (2) pre- and 1-year postoperative long-standing hip-to-ankle radiographs required for CPAK classification and JLCA measurement. Patients with inflammatory arthritis or those who had alignment changes due to ipsilateral diaphyseal fractures or hip arthroplasty were excluded. Accordingly, 323 knees (298 patients) were included.

### Surgical technique and rehabilitation

Surgical planning and the degree of correction were determined based on a long-standing hip-to-ankle radiograph with the patella facing forward. The target weight-bearing point was defined as 62.5% of the tibial plateau width from the medial border, and this was adjusted based on arthroscopic findings that assessed the severity of degenerative changes in each compartment. In cases with advanced cartilage degeneration limited to the medial compartment, the target was maintained or slightly increased, whereas excessive correction was avoided when early degenerative changes were observed in the lateral compartment [18, 19]. For the procedure, the proximal tibial osteotomy site was approached via the anteromedial aspect. After the semitendinosus and gracilis tendons were partially detached, the superficial medial collateral ligament was released distally below the osteotomy level. In all cases, the osteotomy was performed in a supratubercle biplanar manner, with protection of the posterior neurovascular bundles using a blunt retractor. A lateral hinge of approximately 5–9 mm of the lateral cortex was preserved, and the osteotomy site was widened using a laminar spreader under intraoperative fluoroscopy. Then, a fixed-angle plate with locking screws (TomoFix; DePuy Synthes) was placed.

Postoperatively, range of motion exercises were started immediately, with progressive weight-bearing starting with toe-touch during the first two weeks and increasing to full weight-bearing at 4–6 weeks.

**Fig. 1:**
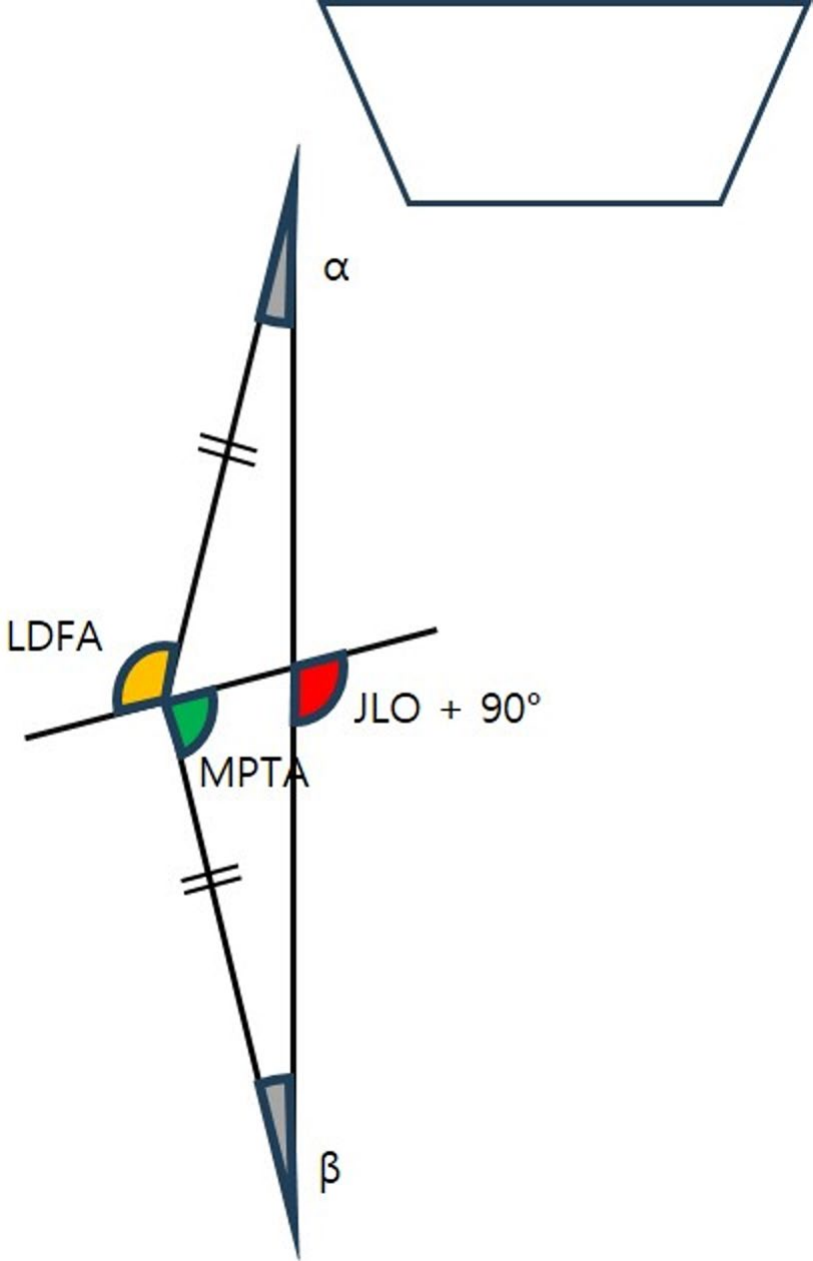
The schematic diagram of the conventional JLO (cJLO) which does not take JLCA into account. Alpha (α) represents the angle between the femoral mechanical axis and the line connecting the centers of the hip and ankle, while beta (β) represents the angle between the tibial mechanical axis and the same line. Assuming equal femoral and tibial lengths (β – α = 0), cJLO can be defined as (LDFA + MPTA)/2–90°. JLO, joint line obliquity; JLCA, joint line convergence angle; LDFA, lateral distal femoral angle; MPTA, medial proximal tibial angle

### Conventional and modified JLOs

JLOs were calculated using two methods based on a geometric approach: cJLO from the CPAK classification and mJLO, which incorporates the JLCA. A geometric analysis was performed under the assumption that the line connecting the centers of the hip and ankle is perpendicular to the ground. Alpha (α) represents the angle between the femoral mechanical axis and the line connecting the centers of the hip and ankle, while beta (β) represents the angle between the tibial mechanical axis and the same line. Then, the cJLO can be expressed as follows through geometric analysis (Fig. 1).

$${\mathrm{cJLO}} + {\mathrm{9}}0^\circ = {\mathrm{LDFA}} - \alpha = {\mathrm{MPTA}} + \beta $$

$${\mathrm{cJLO}} = \left( {{\mathrm{LDFA}} + {\mathrm{MPTA}}} \right)/{\mathrm{2}} + \left( {\beta - \alpha } \right)/{\mathrm{2}} - {\mathrm{9}}0^\circ $$

If the femur and tibia are assumed to be of equal length (β – α = 0), cJLO, defined as (LDFA + MPTA)/2–90°, is identical to the arithmetic JLO defined in the CPAK classification [17]. 

**Fig. 2:**
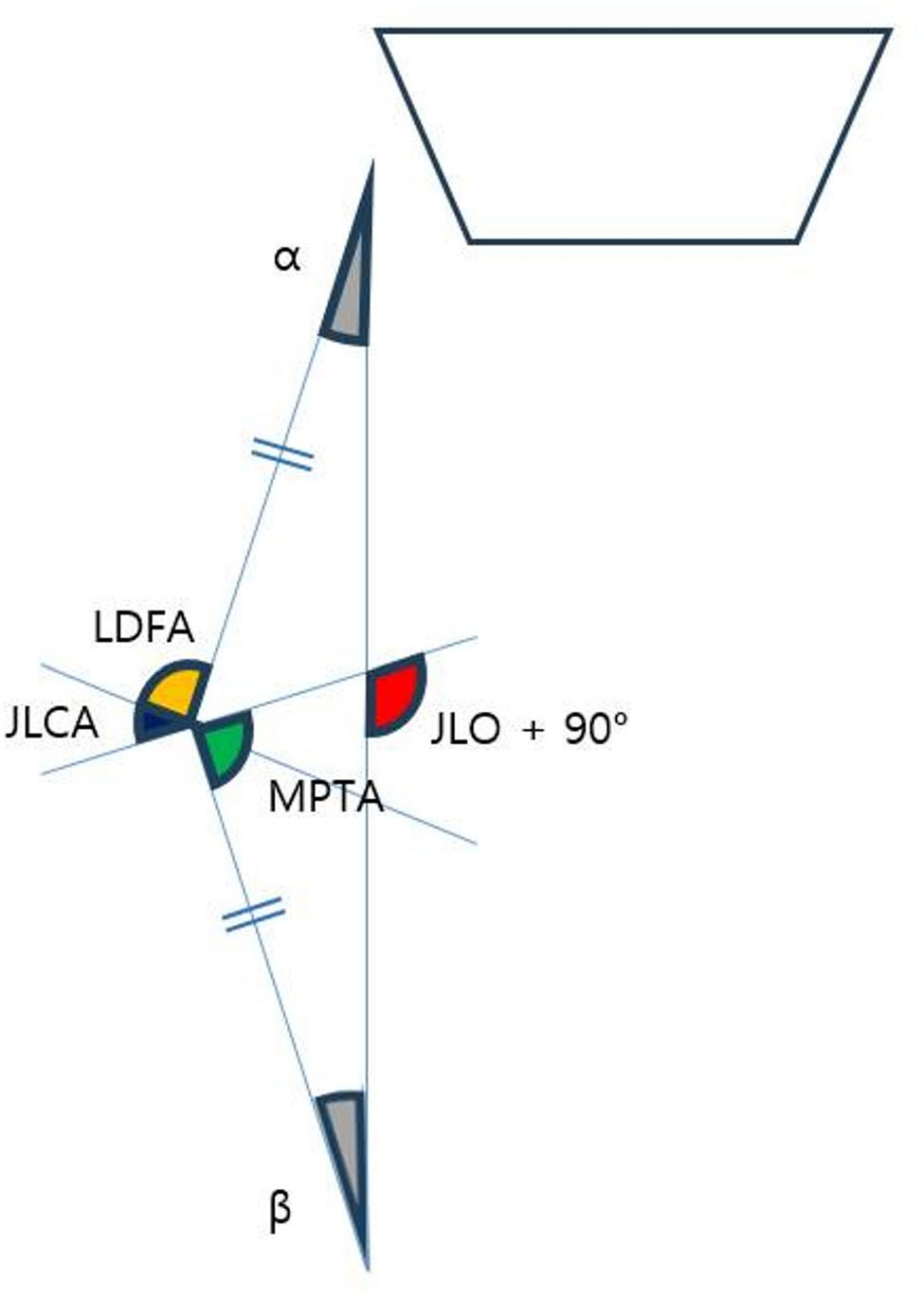
The schematic diagram of the modified JLO (mJLO) which takes JLCA into account. Alpha (α) represents the angle between the femoral mechanical axis and the line connecting the centers of the hip and ankle, while beta (β) represents the angle between the tibial mechanical axis and the same line. Assuming equal femoral and tibial lengths (β – α = 0), mJLO can be defined as (LDFA + MPTA)/2 + JLCA/2–90°. JLO, joint line obliquity; JLCA, joint line convergence angle; LDFA, lateral distal femoral angle; MPTA, medial proximal tibial angle

The mJLO considering JLCA can also be approached using a similar geometric analysis. By rotating the tibial articular line by the JLCA value around the center of the knee joint, the relationship between mJLO and JLCA can be summarized as follows (Fig. 2).

$$ {\mathrm{mJLO}} + {\mathrm{9}}0^\circ = {\mathrm{LDFA}} + {\mathrm{JLCA}} - \alpha = {\mathrm{MPTA}} + \beta $$

$$ \begin{aligned} {\mathrm{mJLO}} = & \left( {{\mathrm{LDFA}} + {\mathrm{MPTA}}} \right)/{\mathrm{2}} + {\mathrm{JLCA}}/{\mathrm{2}} \\ & \quad + \left( {\beta - \alpha } \right)/{\mathrm{2}} - {\mathrm{9}}0^{^\circ } \\ \end{aligned} $$

Under the same assumption (β – α = 0), mJLO can be defined as (LDFA + MPTA)/2 + JLCA/2–90°.

The geometric derivation of the mJLO was based on coronal plane alignment principles under weight-bearing conditions. In long-standing radiographs, the mechanical axis connecting the centers of the hip and ankle was assumed to be perpendicular to the ground, and the femur and tibia were assumed to have comparable lengths, as commonly applied in coronal alignment analyses. Under these assumptions, cJLO represents the arithmetic contribution of femoral and tibial bony alignment components. However, joint line convergence angle (JLCA) reflects intra-articular factors such as cartilage wear and soft tissue laxity, which effectively rotate the tibial articular surface relative to the mechanical axis [12–16]. Geometrically, this intra-articular convergence can be represented as a rotation of the tibial joint line about the center of the knee joint. Because this rotation influences the inclination of the joint line relative to the mechanical axis, half of the JLCA contributes to the apparent JLO when femoral and tibial lengths are assumed to be equal. Accordingly, incorporation of JLCA/2 into the cJLO formula yields a mJLO that more closely reflects the knee joint orientation.

### Radiographic measurements

**Fig. 3:**
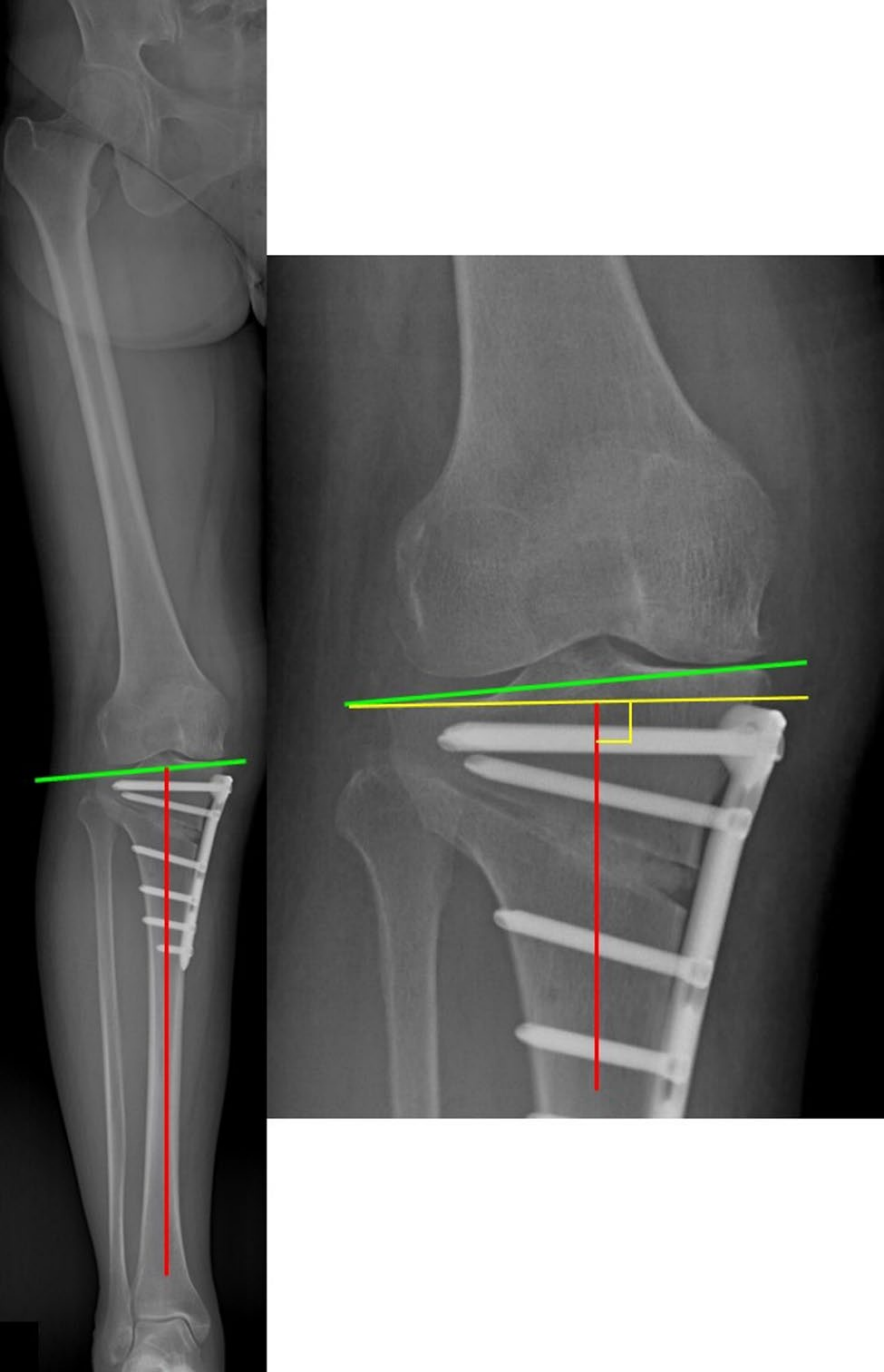
Representative long-standing hip-to-ankle radiograph and magnified knee image demonstrating the knee joint orientation, which was defined as the angle between the tibial articular line (green line) and the horizontal reference line (yellow line). The red line indicates perpendicular line to the ground

Radiographic measurements were obtained from long-standing hip-to-ankle radiographs taken preoperatively and at 1 year postoperatively. The assessed parameters included the mechanical hip-knee-ankle angle (mHKA), mechanical lateral distal femoral angle (LDFA), mechanical proximal tibial angle (MPTA), JLCA, and joint line obliquity (JLO). Knee joint obliquity was defined as the angle between the tibial articular surface and the horizontal plane under weight-bearing conditions (Fig. 3) [5, 6]. Knee phenotypes were categorized based on each JLO method using the modified CPAK classification. Neutral alignment was defined as an mHKA of 0° ± 3°, with varus alignment defined as < − 3° and valgus alignment as > 3°. Neutral JLO was defined as 0° ± 3°, with apex distal JLO defined as > 3° and apex proximal JLO as < − 3°. The same boundary criteria were consistently applied when phenotyping based on each JLO method [17]. 

The calculated cJLO and mJLO values were then compared to the knee joint orientation using intraclass correlation coefficients (ICCs) before and 1 year after OWHTO. Bland-Altman plots were also analyzed to identify which of cJLO or mJLO more accurately reflected the knee joint orientation. Clinical outcomes were evaluated using the Hospital for Special Surgery (HSS) score.

### Statistical analysis

cJLO and mJLO measurements were compared to the knee joint orientation using ICCs and Bland-Altman plots. HSS scores were compared among CPAK classifications using analysis of variance. A sample size estimation was performed based on the comparison of postoperative HSS scores across CPAK classifications, indicating that approximately 35 patients per group would be required to achieve 80% power at a significance level of 0.05. As the primary objective of this study was the methodological validation of the mJLO calculation, the analyses also focused on agreement measures, such as ICCs and Bland–Altman plots. Descriptive statistics for continuous variables are presented as means with standard deviations. All statistical analyses were conducted using R software version 4.4.1 (R Foundation for Statistical Computing, Vienna, Austria), with a significance level set at *p* < 0.05.

**Table 1:** Patients characteristics

	Overall (*n* = 323)		
	Preoperative		Postoperative
Age, y		56.2 ± 7.8	
Male / Female, n		99/222	
BMI, kg/m^2^		26.4 ± 2.8	
LDFA, deg		88.7 ± 1.9	
HKA,^b^ deg	− 7.1 ± 2.7		2.1 ± 2.3
MPTA, deg	84.6 ± 2.1		93.0 ± 2.9
JLCA, deg	3.1 ± 1.9		2.4 ± 1.7
Knee joint orientation,^c^ deg	− 1.0 ± 1.9		1.9 ± 2.3
cJLO,^c^ deg	− 3.3 ± 1.5		0.9 ± 2.0
mJLO,^c^ deg	− 1.8 ± 1.8		2.1 ± 2.3
CPAK classification (cJLO),^d^ n	145 / 54 / 0 / 76 / 48 / 0 / 0 / 0 / 0		0 / 3 / 4 / 3 / 93 / 177 / 0 / 4 / 39
CPAK classification (mJLO),^d^ n	54 / 19 / 0 / 166 / 79 / 0 / 1 / 4 / 0		0 / 1 / 1 / 2 / 98 / 121 / 0 / 12 / 88

BMI, body mass index; LDFA, lateral distal femoral angle; HKA, hip knee ankle angle; MPTA, medial proximal tibial angle; JLCA, joint line convergence angle; JLO, joint line obliquity; CPAK, Coronal Plane Alignment of the Knee

^a^Data are reported as mean ± SD unless otherwise indicated

^b^Positive values indicate valgus alignment whereas negative values indicate varus alignment

^c^Positive values indicate lateral inclination of the joint line

^d^Knee phenotypes (type I / type II / type III / type IV / type V / type VI / type VII / type VIII / type IX) were categorized based on the CPAK classification [17]

## Results

The overall alignment of the study population, measured as the mechanical hip-knee-ankle angle (mHKA), was corrected from − 7.1 ± 2.7° preoperatively to 2.1 ± 2.3° at 1 year postoperatively. Meanwhile, the MPTA increased from 84.6 ± 2.1° to 93.0 ± 2.8°, and the JLCA decreased from 3.1 ± 1.9° to 2.4 ± 1.7°. The HSS scores showed significant improvement, rising from 68.6 ± 11.7 preoperatively to 91.3 ± 7.4 postoperatively (*p* < 0.001). Interobserver reliability for radiographic measurements was excellent, with intraclass correlation coefficients exceeding 0.85 for all key parameters.

In the preoperative phenotype distribution, the most common CPAK type was type IV (*n* = 266, 82.4%), followed by type I (*n* = 37, 11.5%). However, according to the conventional method, the most common types were type I (*n* = 145, 44.9%) and type IV (*n* = 76, 23.5%), while the modified method identified type IV (*n* = 166, 51.4%) and type V (*n* = 79, 24.5%) as the most prevalent. Postoperatively, type V (*n* = 156, 48.3%) was the most common, followed by type VI (*n* = 59, 18.3%). The conventional method identified type VI (*n* = 177, 54.8%) and type V (*n* = 93, 28.8%) as the most common, while the modified method identified type VI (*n* = 121, 37.5%) and type V (*n* = 98, 30.3%; Table 1). In the present cohort, neither conventional nor modified CPAK classifications were associated with significant differences in postoperative HSS scores (conventional CPAK: *p* = 0.304; modified CPAK: *p* = 0.098).

**Table 2:** Intraclass correlation coefficients of cJLO and mJLO

cJLO	Preoperative	Postoperative
	0.842 (0.804–0.873)	0.927 (0.909–0.941)
mJLO	0.881 (0.851–0.904)	0.949 (0.936–0.959)

cJLO, conventional joint line obliquity; mJLO, modified joint line obliquity

^a^The values in parentheses indicate the 95% confidence interval range

## Comparison of agreement with the knee joint orientation between conventional and modified JLOs

**Fig. 4:**
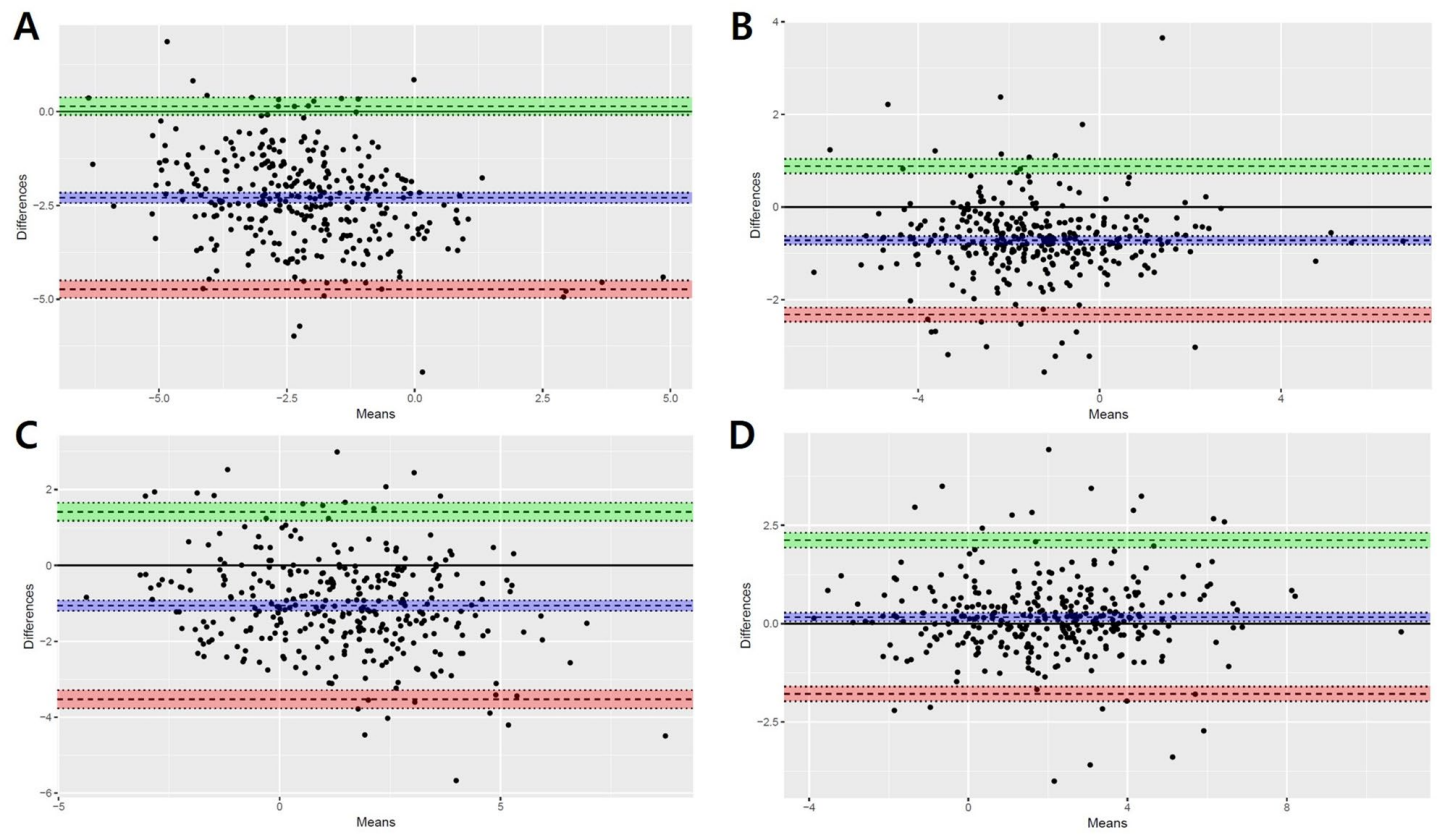
Bland-Altman plots illustrating the consistency between the knee joint orientation and **A** preoperative cJLO, **B** preoperative mJLO, **C** postoperative cJLO, and **D** postoperative mJLO. The blue line represents the mean difference between the two methods, while the green and red lines indicate ± 1.96 standard deviations from the mean difference. The plots show that both (B) preoperative and (D) postoperative mJLOs have smaller differences (− 0.7 ± 1.6° and 0.2 ± 2.2°, respectively) with the knee joint orientation compared to cJLO (− 2.3 ± 2.4° and − 1.0 ± 2.8°, respectively), indicating that mJLO more closely reflected the knee joint orientation. cJLO, conventional joint line obliquity; mJLO, modified joint line obliquity

Knee joint orientation was measured at − 1.0 ± 1.9° preoperatively and 1.9 ± 2.3° at 1 year postoperatively. Comparatively, cJLO values were − 3.3 ± 1.5° and 0.9 ± 2.0°, while mJLO values were − 1.8 ± 1.8° and 2.1 ± 2.3° (Table 1). When examining the agreement of the two methods through ICCs, preoperative cJLO showed an ICC of 0.842 (95% confidence interval [CI], 0.804–0.873), and postoperative 1-year cJLO had an ICC of 0.927 (95% CI, 0.909–0.941). However, preoperative mJLO demonstrated an ICC of 0.881 (95% CI, 0.851–0.904), and postoperative 1-year mJLO achieved an ICC of 0.949 (95% CI, 0.936–0.959; Table 2). Bland-Altman plots further supported these findings, showing that both preoperative and postoperative 1-year mJLOs had smaller differences (− 0.7 ± 1.6° and 0.2 ± 2.2°, respectively) with the knee joint orientation compared to cJLOs (− 2.3 ± 2.4° and − 1.0 ± 2.8°, respectively), indicating that mJLOs more closely reflected the joint line orientation (Fig. 4).

## Discussion

The primary finding of the present study was that the CPAK classification can be applied to OWHTO patients, but that JLCA should be considered when calculating the JLO. The formula accounting for JLCA was derived through a geometric approach. The resulting mJLO, expressed as (LDFA + MPTA)/2 + JLCA/2° − 90°, was found to more closely reflect the knee joint orientation compared to cJLO. Considering both the surgical target (mHKA), and the important prognostic factor (JLO), the CPAK classification can be effectively applied to categorize knee phenotypes in OWHTO. From a clinical perspective, incorporating JLCA into JLO calculation may improve radiographic phenotyping and aid in anticipating excessive JLO during alignment correction planning, particularly in knees with substantial preoperative JLCA, in which conventional calculations tend to underestimate JLO. In addition, mJLO-based phenotyping may facilitate more consistent interpretation of postoperative alignment changes and outcomes assessments.

The CPAK classification has provided valuable insights into reconstructive knee surgery [10, 20]. Given that JLO is a critical parameter for determining surgical planning and predicting prognosis in OWHTO, the CPAK classification is expected to have significant utility in this procedure as well [19, 21]. However, since adequate release of the superficial medial collateral ligament is a prerequisite for OWHTO, changes in JLCA are inevitable [14, 22, 23], a factor not accounted for in the original CPAK classification. While a previous study attempted to improve the accuracy of arithmetic JLO [17], the present study revealed that the cJLO, without considering JLCA, showed discrepancies when compared to the knee joint orientation.

This study quantified the impact of the JLCA on JLO. Using a geometric approach, it demonstrated that incorporating half the JLCA value into the conventional JLO closely aligns with the knee joint orientation, as confirmed by ICCs and Bland-Altman plots. Preoperative ICCs for cJLO and mJLO were 0.842 (95% CI, 0.804–0.873) and 0.927 (95% CI, 0.909–0.941), respectively, compared to postoperative ICCs of 0.881 (95% CI, 0.851–0.904) and 0.949 (95% CI, 0.936–0.959). This indicates a greater preoperative discrepancy, likely due to the more significant influence of JLCA before correcting varus malalignment. These findings underscore the critical role of JLCA in JLO calculations. A similar trend was observed in Bland-Altman plots. Although the mean difference from the knee joint orientation was lower for mJLO compared to cJLO, the overall difference was more pronounced preoperatively than postoperatively (− 0.7 ± 1.6° vs. − 2.3 ± 2.4°, preoperative; 0.2 ± 2.2° vs. − 1.0 ± 2.8°, postoperative). Considering that the impact of JLCA omission is more significant in cases of malalignment, the application of CPAK classification in OWHTO should be based on the premise of accounting for JLCA.

To avoid conceptual ambiguity, it is important to distinguish among several related but distinct parameters describing knee joint morphology. Knee joint orientation was defined as the angle between the tibial articular line and a horizontal ground reference on long-standing hip-to-ankle radiographs, and was used as a clinical reference to assess how well computed indices approximate joint line orientation in OWHTO. The cJLO, calculated as 90° − (LDFA + MPTA)/2, attempts to translate bony configuration into a physical inclination of the joint line but does not account for intra-articular convergence caused by cartilage wear or soft tissue laxity. The mJLO further incorporates half of the JLCA, thereby integrating intra-articular and soft tissue–related factors into the estimation of joint line inclination. From a biomechanical perspective, this modification allows mJLO to better reflect the functional joint line orientation experienced during weight bearing. Clinically, this modification is particularly relevant in OWHTO, where changes in JLCA following ligament release and deformity correction can substantially alter postoperative knee joint orientation. In this context, mJLO should be interpreted as a refinement within the CPAK framework, rather than a replacement of the original classification, as it incorporates intra-articular factors to better approximate functional joint orientation.

When examining the knee phenotype distribution according to each JLO calculation method, a few aspects warrant attention. Preoperatively, 38.4% of knees were classified as having a horizontal joint line (types IV and V) using cJLO, whereas knee joint orientation showed that 86.1% had a horizontal joint line. The modified method (mJLO) identified 75.9% as types IV and V. Similarly, after OWHTO, 84.5% and 65.3% of knees were estimated to have a horizontal joint line (types IV, V, and VI) based on cJLO and mJLO, respectively, while the knee joint orientation indicated 67.8%. These discrepancies according to JLO calculation methods can be attributed to the influence of JLCA, which induces a compensatory horizontal shift in JLO. Another consideration is that, despite OWHTO being performed to address varus malalignment originating from the joint (represented by JLCA) or proximal tibia (represented by MPTA), the preoperative distribution was shaped by defining the neutral JLO boundaries as 0 ± 3°. To improve the applicability of the CPAK classification in OWHTO, narrowing these boundaries may be necessary.

Recent studies have further highlighted the limitations of the CPAK classification as a two-dimensional alignment system [24–26]. CPAK classification has been shown to have limited ability to capture segmental or extra-articular deformities, which may contribute to discrepancies between radiographic classification and the underlying anatomical or biomechanical conditions of the knee [26]. In addition, it has been reported that CPAK classification does not consistently identify the true joint apex position and may not reliably predict clinical or radiological outcomes in certain osteotomy settings [25]. These findings suggest that reliance on bony alignment parameters alone may be insufficient to fully characterize knee joint orientation. In this context, incorporation of intra-articular factors such as JLCA may provide a more comprehensive representation of knee joint orientation, particularly in conditions where soft tissue laxity and cartilage wear substantially influence alignment.

From a clinical perspective, mJLO may provide a closer representation of functional joint orientation, which could have potential implications for surgical planning and postoperative evaluation in OWHTO. Conventional arithmetic JLO calculations tend to underestimate joint orientation in knees with substantial preoperative JLCA. By incorporating JLCA, mJLO may better reflect functional alignment, particularly in such cases. The benefit of incorporating JLCA appears to be most relevant in varus CPAK phenotypes characterized by substantial intra-articular convergence. In these knees, conventional arithmetic JLO tends to underestimate joint orientation, whereas mJLO more closely reflects functional alignment. However, these potential implications should be interpreted with caution, and further longitudinal studies are needed to determine whether mJLO-guided planning or phenotype-based strategies can improve long-term outcomes or reduce the risk of alignment-related complications after OWHTO.

Several limitations should be noted. First, measurement errors might have occurred during the analysis of radiographic parameters. Such risk was mitigated by having two orthopedic surgeons independently conduct the measurements, remaining unaware of each other’s results. Although excellent interobserver reliability was observed, measurement error inherent to radiographic assessment cannot be entirely excluded. Second, the potential influence of the hip and ankle joints on JLO was not analyzed [5, 27]. Exploring these compensatory mechanisms could offer a more comprehensive understanding of JLO. Third, the radiologic parameters were assessed using only a 2-dimensional plane. While the CPAK classification is originally designed to intuitively represent knee phenotypes in the coronal plane, it may have limitations when evaluating the complex 3-dimensional knee joint structures. Fourth, although a sample size estimation was performed and the overall cohort included 323 knees, some CPAK subtypes comprised relatively small numbers of patients, which may have limited the statistical power for comparisons across individual phenotypes.

## Conclusion

The mJLO, derived through a geometric approach that incorporates JLCA, was found to more closely reflect the knee joint orientation compared to the cJLO described in the CPAK classification. To effectively apply the CPAK classification in OWHTO, it is essential to account for JLCAs before and after surgery. Accordingly, mJLO can be represented as (LDFA + MPTA)/2 + JLCA/2° − 90°.

## Data Availability

No datasets were generated or analysed during the current study.
